# Validity of the Global Physical Activity Questionnaire (GPAQ) in assessing levels and change in moderate-vigorous physical activity and sedentary behaviour

**DOI:** 10.1186/1471-2458-14-1255

**Published:** 2014-12-10

**Authors:** Claire L Cleland, Ruth F Hunter, Frank Kee, Margaret E Cupples, James F Sallis, Mark A Tully

**Affiliations:** UKCRC Centre of Excellence for Public Health (NI), Centre for Public Health, School of Dentistry, Medicine and Biomedical Sciences, Queen’s University Belfast, Clinical Sciences Block B, Royal Victoria Hospital, Grosvenor Road, Belfast, BT12 6BJ Northern Ireland; MRC/CSO Social and Public Health Sciences Unit, University of Glasgow, Top floor, 200, Renfield Street, Glasgow, G2 3QB Scotland; Department of General Practice and Primary Care, School of Dentistry, Medicine and Biomedical Sciences, Queen’s University Belfast, 1, Dunluce Avenue, Belfast, BT9 7HR Northern Ireland; Department of Family and Preventive Medicine, University of California, San Diego, USA

**Keywords:** GPAQ, Validation, Measurement, Physical activity, Accelerometer, Sedentary behaviour

## Abstract

**Background:**

Feasible, cost-effective instruments are required for the surveillance of moderate-to-vigorous physical activity (MVPA) and sedentary behaviour (SB) and to assess the effects of interventions. However, the evidence base for the validity and reliability of the World Health Organisation-endorsed Global Physical Activity Questionnaire (GPAQ) is limited. We aimed to assess the validity of the GPAQ, compared to accelerometer data in measuring and assessing change in MVPA and SB.

**Methods:**

Participants (n = 101) were selected randomly from an on-going research study, stratified by level of physical activity (low, moderate or highly active, based on the GPAQ) and sex. Participants wore an accelerometer (Actigraph GT3X) for seven days and completed a GPAQ on Day 7. This protocol was repeated for a random sub-sample at a second time point, 3–6 months later. Analysis involved Wilcoxon-signed rank tests for differences in measures, Bland-Altman analysis for the agreement between measures for median MVPA and SB mins/day, and Spearman’s rho coefficient for criterion validity and extent of change.

**Results:**

95 participants completed baseline measurements (44 females, 51 males; mean age 44 years, (SD 14); measurements of change were calculated for 41 (21 females, 20 males; mean age 46 years, (SD 14). There was moderate agreement between GPAQ and accelerometer for MVPA mins/day (r = 0.48) and poor agreement for SB (r = 0.19). The absolute mean difference (self-report minus accelerometer) for MVPA was −0.8 mins/day and 348.7 mins/day for SB; and negative bias was found to exist, with those people who were more physically active over-reporting their level of MVPA: those who were more sedentary were less likely to under-report their level of SB. Results for agreement in change over time showed moderate correlation (r = 0.52, p = 0.12) for MVPA and poor correlation for SB (r = −0.024, p = 0.916).

**Conclusions:**

Levels of agreement with objective measurements indicate the GPAQ is a valid measure of MVPA and change in MVPA but is a less valid measure of current levels and change in SB. Thus, GPAQ appears to be an appropriate measure for assessing the effectiveness of interventions to promote MVPA.

## Background

Non-communicable chronic diseases (NCDs) are the leading causes of morbidity and mortality in both the developing and developed nations [1–3]. Specifically, within the United Kingdom (UK) it has been reported by the World Health Organisation (WHO) that since 2000 they account for 89% of total deaths and the most common NCDs in order of prevalence are: cardiovascular diseases, cancers, chronic respiratory diseases and diabetes [4]. These diseases are associated with a variety of determinants including physical inactivity, poor nutritional choices, tobacco use and socio-economic status [1, 2, 5]. Of note ‘physical inactivity’ describes when some form of physical activity is performed but not to a level that would produce a distinct increase in levels of energy expenditure [6].

In addition, more recent attention has focused on the health consequences of sedentary behaviour (SB) as a distinct behaviour, in relation to the development of NCDs [7]. Sedentary behaviour is often used synonymously with ‘physical inactivity’ although it should be noted that it has been defined differently as “activities that include energy expenditure at a level of 1.0-1.5 metabolic equivalent units (MET)”; for reference, sitting equates to 1.0 MET [8]. Sedentary behaviour is commonly measured as time spent sitting (e.g. computer use, working, watching television, reading) and is considered as a risk factor for cardiovascular and metabolic disease, independent of time spent in physical activity (PA) [8–11]. To ensure clarity for public health-related research, PA has been defined as “any bodily movement produced by skeletal muscles that results in energy expenditure”; this includes activities of daily life in work, leisure time, during employment or in the house [12]. Physical activity can be classified into three intensities: light (1.6-2.9 MET e.g. slow walking, household chores); moderate (3.0-6.0 MET e.g. walking, golf, light cycling, dancing); and vigorous (>6.0 MET e.g. football, tennis, jogging/running, boxing) [13].

Both PA and SB have been recognised as having a vital role in the prevention and treatment of NCDs [13]. As both PA and SB are health priorities, it is important to measure the behaviours with high quality instruments in both public health practice and research, to provide evidence for informed policy decision making [1, 2, 14, 15]. Considering the potential significance that PA and SB have for health, the measurement of both behaviours must be performed using valid and reliable measurement tools which are consistent on a local, national and international scale to allow meaningful comparisons of behaviour among populations and to determine the effectiveness of behaviour change interventions [1, 15–17].

The Global Physical Activity Questionnaire (GPAQ) was developed in 2002 by the World Health Organisation (WHO) as part of the WHO STEPwise Approach to Chronic Disease Risk Factor Surveillance for PA observation [18]. Its use in national surveillance of PA was recommended by the WHO in their 2004 *Global Strategy on Diet, Physical Activity and Health*[19]. The GPAQ consists of 16 questions designed to estimate an individual’s level of PA in 3 domains (work, transport and leisure time) and time spent in SB [15, 20]. Taking into consideration that the GPAQ was initially developed as a surveillance tool, to be used for evaluation and comparison of PA levels on both a local and international scale, it is important that the limited evidence base for its validity is further developed, particularly for use in behaviour change interventions [21, 22].

GPAQ has been previously assessed in terms of its validity and reliability in a nine country study implemented by Bull et al., in 2009, and it was more recently validated in Malaysian, Vietnamese and American adults [15, 22–25]. Evidence for the validity of the GPAQ in European countries is somewhat lacking and requires further investigation as it may be influenced by cultural norms, levels of education, and differences in perceived social desirability [26]. Previous studies showed that the criterion-related validity of the GPAQ for moderate intensity PA, as determined by an accelerometer, was poor (South Africa r = −0.03) and fair (China r = 0.23) (15); comparison of the GPAQ and accelerometer data for vigorous PA showed fair criterion-related validity (South Africa r = 0.26 and China r = 0.23) (15). With most prior studies being conducted in low- to moderate-income countries where low education could have contributed to lower validity than expected, GPAQ should be further evaluated in high-income countries. Moreover, none of the studies referenced have assessed the validity of the GPAQ in measuring changes in PA, despite its proposed uses.

Therefore, in a sample of adults residing in the UK the aims of the current study were to:

1. Assess the criterion validity of the GPAQ compared to accelerometer data in determining moderate-to-vigorous physical activity (MVPA) and time spent in SB.
2. Assess the validity of the GPAQ when estimating changes in PA and SB over time.

## Methods

The study was approved by the Office for Research Ethics Committees, Northern Ireland (09/NIR02/66).

### Participant recruitment

Participants were selected randomly from those who consented to take part in further research, following their completion of the 2010/2011 Physical Activity and the Rejuvenation of Connswater (PARC) Study Household Survey [26]. For the current study, the aim was to recruit a sample of 100 individuals with an equal distribution across sexes and levels of PA, which, according to Bland and Altman [27], would allow for 95% confidence intervals of approximately +/−0.34 standard deviations.

Participants were selected using computer based random number generation, with stratification by sex and by level of PA (high, moderate or low), as determined by the GPAQ which was a component of the PARC Household Survey, using the GPAQ Analysis Guide [18].

**Figure 1:**
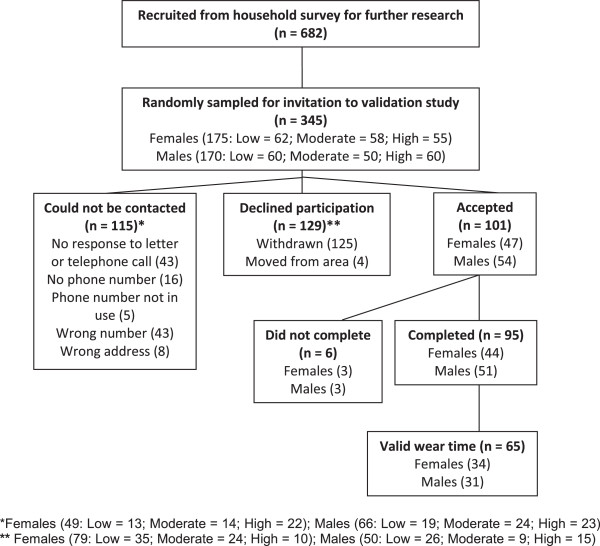
**Flow diagram for participants in validation study (Time 1).**

The PA level of each participant was measured between May and October 2011, and a random sub-sample (again selected using computer based random number generation) had their measurements repeated between November and December 2011 (Figure 1). All participants provided written informed consent prior to commencing both studies.

### Measures of physical activity

At the beginning of each measurement period, study participants were visited at their home, where the researcher explained the purpose of the study and demonstrated the use of the Actigraph GT3X accelerometer (Actigraph Inc., Florida, US). Participants were asked to wear the accelerometer on their hip for seven consecutive days and afterwards to complete a GPAQ. Activity counts were recorded in one second epochs. In order to obtain total minutes of MVPA per day, the data were reintegrated in 60 second epochs before Freedson cut off points were applied to the data: sedentary (≤ 100 counts min^−1^), light (101–1951 counts min^−1^), moderate (1952–5724 counts min^−1^) and vigorous PA (≥ 5725 counts min^−1^) [28, 29].

Non-wear time was defined as a run of zero counts lasting more than 60 minutes [30]. At least five valid days (including one weekend day) were required for inclusion in the analysis; a valid day was defined as a 24-hour period in which more than 600 minutes of wear time were recorded [31, 32]. At the end of the study period, the researcher collected the accelerometer and invited participants to complete the GPAQ again.

### Data management

The GPAQ data were entered manually into SPSS Data Analysis Version 17.0 (SPSS Inc, Chicago, IL). Ten percent of data were checked for accuracy (by CLC) of entry and results showed 100% accuracy. The GPAQ data were cleaned and processed using the WHO Steps programme [18]. Accelerometer data were processed using ActiLife version 5.2.0. For the purpose of this study both the GPAQ and accelerometer data were reported as continuous variables (min·d^−1^).

### Data analysis

Statistical analysis was performed using SPSS Data Analysis Version 17.0. Independent t-tests were performed to compare baseline demographic variables of non-participants and participants. Kolmogorov-Smirnov tests confirmed that the GPAQ data were not normally distributed (p < 0.05), and data are therefore presented as medians and inter-quartile range. Differences in the measurements between instruments of 1) median minutes of MVPA per day and 2) median minutes of SB per day were assessed using Wilcoxon-signed rank tests.

Using data from time 1 (T1), criterion-related validity was assessed by comparing minutes of MVPA and SB per day, as measured by GPAQ, to accelerometer-determined data, using Spearman’s rho coefficient. Previous research has identified sex differences in levels of MVPA [33]. We therefore also repeated the analysis for males and females separately, to determine if any differences in validity existed between sexes.

Bland-Altman analysis was performed during the current study to assess agreement between reported and objective measures of PA for both minutes of MVPA per day and minutes of SB per day [27, 34]. Bland-Altman plots were produced using the formulas:

1. Mean [GPAQ minutes of MVPA/day + accelerometer minutes of MVPA/day]/2 and;
2. Difference [GPAQ minutes of MVPA/day - accelerometer minutes of MVPA/day].

Limits of agreement were also calculated with the accelerometer data: Mean difference between instruments (GPAQ minus accelerometer) ± (1.96 × standard deviation).

The extent of change from T1 to time 2 (T2) was assessed as the difference between measures at T1 minus T2. Spearman’s rho coefficients were calculated to assess agreement between the change scores derived from the two instruments.

To interpret the Spearman’s rho coefficient we used the following benchmarks: 0–0.20 = poor correlation, 0.21-0.40 = fair correlation, 0.41-0.60 = moderate/acceptable correlation, 0.61-0.80 = substantial correlation, 0.81-1.0 = near perfect correlation [35]. Significance was determined at the level of *p* <0.05.

## Results

### Demographic characteristics

Of the 345 participants selected to participate, we were unable to make telephone or postal contact with 115; 129 declined participation (moved house, pregnant, currently sick, no longer interested). Thus a response rate of 44% (101/230) for potential participants who were contacted was achieved for the validation study.

There were no statistically significant differences between participants and those who declined to participate in age, sex and minutes of MVPA/day (*p >* 0.05). Of the 101 participants, 6 were excluded from analysis (3 females; 3 males) for either not wearing the accelerometer for the required period of time (i.e. < 7 days) (n = 3) or failing to fully complete the questionnaires (n = 3) (Figure 1). Of the 95 participants who returned data at the end of T1, 44 were female and 51 were male; their mean age was 44 years (SD 14). Of these 95 participants, only 65 (34 female; 31 male) had valid accelerometer wear time (worn for 600 minutes each day for at least five of the seven consecutive study days, including one weekend day); their mean age also was 44 years (SD 14) (Table 1).

**Figure 2:**
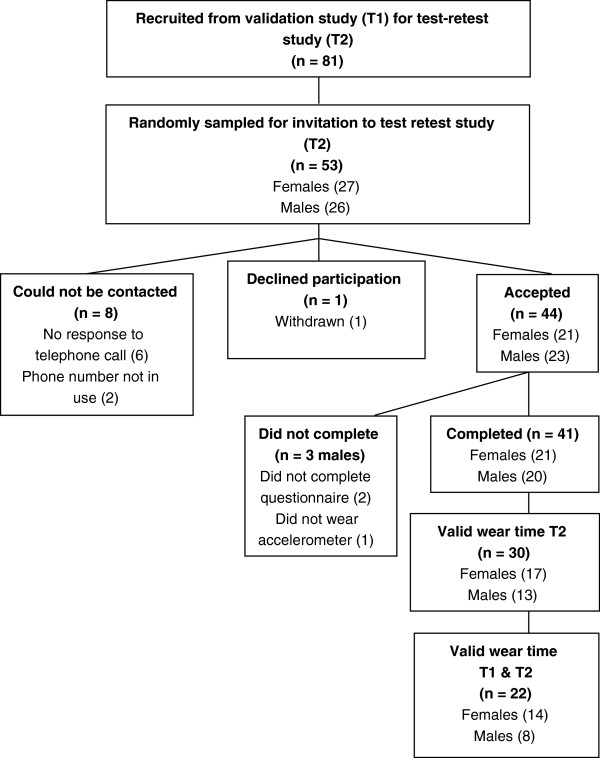
**Flow diagram for participants in test retest study (Time 2).**

Further contact by the research team for the T2 measurements was agreed by 81 of the 101 participants. Of those, 53 (27 females; 26 males) were selected randomly to participate in the follow-up study, but we were unable to contact 8 participants (Figure 2). Of the 45 who were contacted, 98% (44/45) agreed to complete T2 measurements; 1 individual declined participation when re-contacted. Of these 44, 3 males failed to provide a complete dataset by either not wearing the accelerometer for the required period of time (n = 1) or not fully completing the questionnaire (n = 2). Of those who completed the test-retest study (n = 41), 21 were female and 20 were male, with a mean age of 46 years (SD 14).

Of the 41 participants who completed the second phase of the study 30 (17 female; 13 male; mean age 48 years (SD13)) had valid accelerometer wear time at T2 (wore the monitor for 600 minutes each day for at least five days, including one weekend day). However, 8 of these participants did not have valid accelerometer wear time at T1, so assessing the extent of change was possible only for 22 (Figure 2).

**Table 1:** **Demographic characteristics of participants recruited to the validation (Time 1) and follow-up (Time 2) studies**

	Time 1		Time 2	
	Overall sample	Valid sample*	Overall sample	Valid sample*
	(n = 95)	(n = 65)	(n = 41)	(n = 30)
***Sex***	**N (%)**	**N (%)**	**N (%)**	**N (%)**
*Female*	44 (46)	34 (52)	21 (51)	17 (57)
*Male*	51 (54)	31 (48)	20 (49)	13 (43)
***Age (years), mean (SD)***				
Overall sample	44 (14)	44 (14)	46 (14)	48 (13)
*Female*	43 (13)	44 (14)	45 (13)	49 (12)
*Male*	45 (15)	45 (13)	46 (15)	48 (14)
Physical activity level**	**N (%)**	**N (%)**	**N (%)**	**N (%)**
Low	25 (26)	18 (28)	12 (29)	9 (30)
*Females*	11	10	7	5
*Males*	14	8	5	4
Moderate	35 (37)	27 (41)	15 (37)	12 (40)
*Females (N)*	19	14	8	8
*Males (N)*	16	13	7	4
High	35 (37)	20 (31)	14 (34)	9 (30)
*Females (N)*	15	10	6	4
*Males (N)*	20	10	8	5

*Participants who had valid accelerometer wear time (5 days including one weekend day).

**As reported by GPAQ in previously administered household survey [24].

### Assessment of MVPA

Comparing data from the two instruments at T1 (n = 65) showed that the median minutes of MVPA measured by the GPAQ was 30 minutes compared to 56 minutes measured by the accelerometer. This difference was not statistically significant overall (*p* = 0.073); it reached statistical significance for females (p = 0.048), but not for males (p = 0.544) (Table 2).

**Table 2:** **Assessment of minutes of MVPA per day and time spent in sedentary behaviour per day as measured by GPAQ and accelerometer at Time 1**

Measurement tool	Median MVPA per day (mins)	Inter quartile range	***T*** -test of difference	Median sedentary time per day (mins)	Inter quartile range	***T*** -test of difference
**Validation study (n = 65)**						
GPAQ	30	17-75	*p =* 0.073	300	180-435	*p =* 0.0001
Accelerometer	56	44-74		696	617-751	
**Females (n = 34)**						
GPAQ	30	15-62	*p* = 0.048	240	180-360	*p* = 0.0001
Accelerometer	49.5	44-66		660	613-719	
**Males (n = 31)**						
GPAQ	43	19-120	*p* = 0.544	360	180-480	*p* = 0.0001
Accelerometer	65	48-76		715	627-776	

A moderate level of agreement between the GPAQ and accelerometer data for MVPA at T1 (n = 65) was observed for criterion validity (r = 0.484; *p* < 0.005). Sub-group analysis by sex showed similar moderate correlations for criterion validity for females (r = 0.434; *p* = 0.010) and males (r = 0.496; *p* = 0.005) (Table 3).

**Figure 3:**
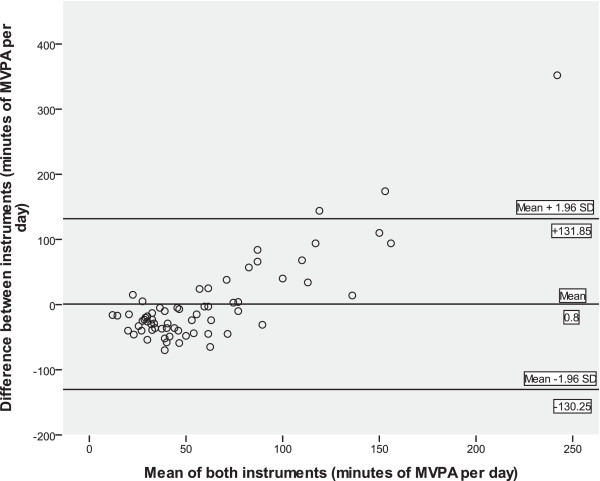
**Bland-Altman plots of minutes of MVPA per day between the GPAQ self-report questionnaire and Actigraph GT3X accelerometer.**

Results for the Bland-Altman analysis showed that the difference between the two instruments was 0.8 minutes of MVPA per day (SD 66.86). The limits of agreement for the two instruments were wide, with the difference lying between −130.25 and +131.85 mins/day (Figure 3). Following a review of Figure 3 it would appear that negative bias exists for the GPAQ with the majority of points falling below zero. In addition, those people who were more physically active were found to be more likely to over-report their level of physical activity using the GPAQ (Figure 3).

**Table 3:** **Moderate-to-vigorous physical activity (mins/day): Spearman’s rho coefficient correlations for the GPAQ vs accelerometer**

Sample	n	Variables	Time point	Spearman’s rho coefficient (r)	***p*** value
**Criterion validity**					
Overall sample*	65	GPAQ vs. Accelerometer (mins/day MVPA)	Time point 1	0.484	0.000
Females	34			0.434	0.010
Males	31			0.496	0.005
**Extent of change**					
Overall sample**	22	Change between T1 and T2:	Time point 1 vs. Time point 2	0.523	0.12
		GPAQ vs. accelerometer (mins/day MVPA)			

*Overall sample that had valid accelerometer wear time (validation study; T1) (65/95).

**Overall sample that had valid accelerometer wear time (T1 and T2) (22/41).

MVPA: moderate-to-vigorous physical activity.

The mean (±SD) change in MVPA over the two time points was 3.55 ± 64.60 mins/day (range −111 mins/day to 223 mins/day) when measured by the GPAQ and −3.05 ± 16.62 mins/day (range −33 mins/day to 47 mins/day) when measured by the accelerometer. Comparing the extent of change in MVPA over the two time points (n = 22) indicated that there was a moderate level of agreement between the instruments (r = 0.523; *p* = 0.12) (Table 3).

### Assessment of SB

The median minutes of SB from T1 (n = 65) was 300 mins/day as measured by the GPAQ and 696 mins/day from the accelerometer. This difference was significant for the overall group (*p* = 0.0001), females (p = 0.0001) and males (p = 0.0001) (Table 2).

A non-significant, poor level of agreement between the GPAQ and accelerometer data for SB at T1 (n = 65) was observed (r = 0.187; p = 0.135). Sub-group analysis by sex showed fair level correlations for females (r = 0.378; p = 0.027) but a poor correlation for males (r = −0.053; p = 0.778) (Table 4).

**Figure 4:**
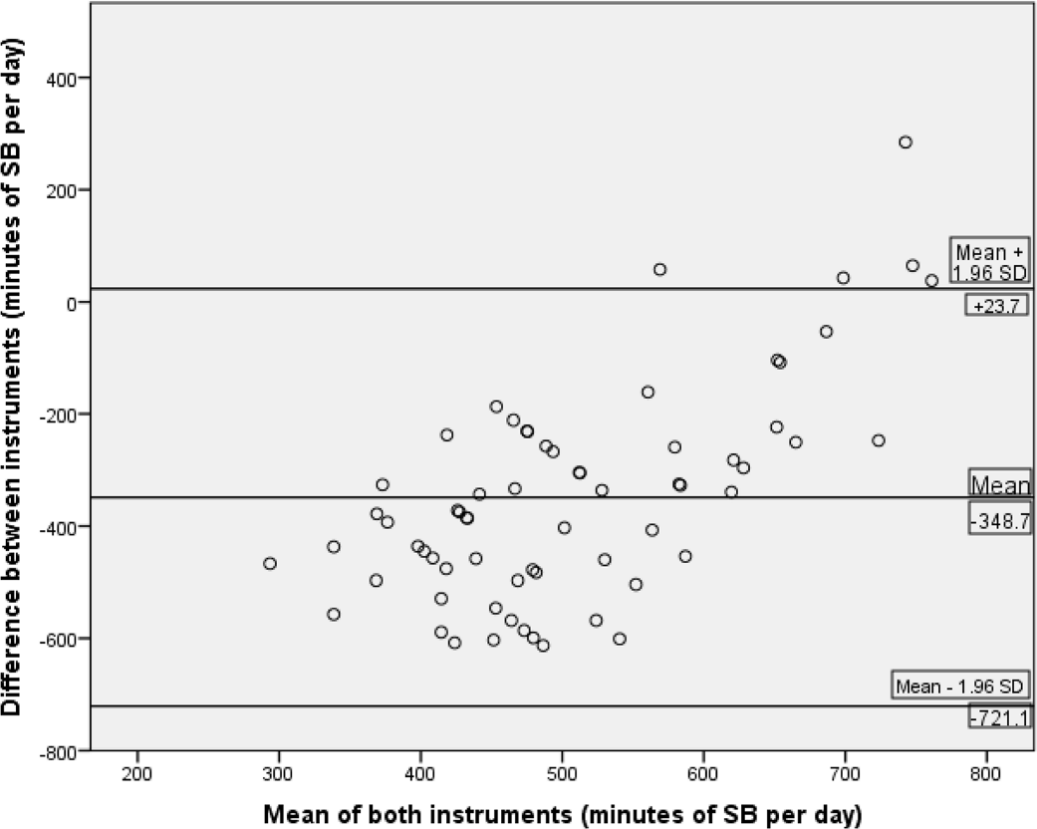
**Bland-Altman plots of minutes of sedentary behaviour per day between the GPAQ self-report questionnaire and Actigraph GT3X accelerometer.**

Results for the Bland-Altman analysis showed that the difference between the two instruments was −348.7 minutes of SB per day (SD 190). Limits of agreement for the two instruments (GPAQ and accelerometer) were wide, with the difference lying between −721.10 and +23.70 mins/day (Figure 4). Following a review of Figure 4 it would appear that negative bias exists for the GPAQ with the majority of points falling below zero. In addition, following a review of Figure 4 it would appear that bias exists, and those people who were found to be more sedentary were less likely to under-report their level of sedentary behaviour using the GPAQ (Figure 4).

**Table 4:** **Sedentary behaviour (min/day): Spearman’s rho coefficient correlations for the GPAQ vs accelerometer**

Sample	n	Variables	Time point	Spearman’s rho coefficient (r)	***P*** value
**Criterion validity**					
Overall sample*	65	GPAQ vs. Accelerometer (mins/ day)	Time point one	0.187	0.135
Females	34			0.378	0.027
Males	31			−0.053	0.778
**Extent of change**					
Overall sample**	22	Change between T1 and T2:	Time point one vs. Time point two	−0.024	0.916
		GPAQ vs. accelerometer (mins/day SB)			

*Overall sample that had valid accelerometer wear time (validation study; T1) (65/95).

**Overall sample that had valid accelerometer wear time (T1 and T2) (22/41).

The mean (±SD) change in SB over the two time points was 27.73 ± 137.94 mins/day (range −270 mins/day to 210 mins/day) when measured by the GPAQ and 20.50 ± 75.22 mins/day (range −177 mins/day to 133 mins/day) when measured by the accelerometer. Comparing the extent of change in SB over the two time points (n = 22) indicated a poor level of agreement between the instruments (r = −0.024; *p* = 0.916) (Table 4).

## Discussion

### Validity of GPAQ assessment of MVPA

The results demonstrate moderate evidence of criterion-related validity for the GPAQ when compared with accelerometer minutes of MVPA (r = 0.484). The levels of correlation found in our sample (UK) and those found by Herrmann et al. [22] (USA r = 0.26) were substantially higher than the highest correlations found for moderate PA in low-income countries reported by Bull et al. [15] (South Africa (r = −0.03) and China (r = 0.23)). Higher validity of GPAQ in higher-income countries is likely due to high education levels. Some discrepancies can occur between self-report measures and accelerometers, as accelerometers do not register all upper body movements e.g. swimming and cycling [17]. This could be an issue when comparing findings from the UK and USA with results from South Africa and China, because work related PA in the latter countries is more commonly associated with upper body movements [16, 23]. Present validity estimates for GPAQ were slightly higher than estimates based on similar methods with the widely used International Physical Activity Questionnaire (IPAQ) [29].

Although GPAQ had a moderate correlation with accelerometer for minutes of MVPA per day, the inter-quartile range was wide for the GPAQ in comparison to the narrower range found for the accelerometer. Therefore, at the level of the individual where a precise measurement may be required, the GPAQ may not offer a desirable degree of precision, meaning that GPAQ may not be an appropriate tool for assessing an individual’s level of PA. This finding concurs with the recommendation by Ekelund et al. who suggested that self-reported physical activity in general was not sufficiently accurate for individual assessment [36].

A relatively novel finding was the moderate correlation between the GPAQ and accelerometer for the extent of change in minutes of MVPA/day. Though PA self-reports are widely used to evaluate interventions, they are rarely validated in their ability to detect change. Therefore, whilst the GPAQ might not be considered an appropriate tool to measure the effectiveness of a physical activity intervention at the level of the individual, it would be acceptable to measure effectiveness at the level of a community or population, using minutes of MVPA/day as the outcome measure.

### Validity of GPAQ assessment of SB

The results of the current study showed that a significantly higher level of SB was measured by the accelerometer than was self-reported by participants using the GPAQ. This finding is similar to that of a recent study by Clemes et al. who found that when a self-report measure of SB was comprised of a single SB item (true for the GPAQ) it significantly underestimated SB in comparison to accelerometer data [37]. Our finding of a poor correlation (r = 0.187) between GPAQ measurement for minutes of SB per day and accelerometer data concurs with previous studies [38], though validity findings with the IPAQ sitting items were higher for both long (r = 0.33) and short (r = 0.34) forms [39].

Developing accurate and valid methods of SB measurement is important for this growing area of research, both for epidemiological and intervention studies [40]. Measuring SB is challenging, and more research is needed particularly when aiming to distinguish between behaviours such as lying down, sitting or standing [37, 40–42].

Present findings demonstrate that GPAQ may not be appropriate when assessing minutes of SB or SB change after an intervention in an individual or a population. More accurate measurement of SB may be provided by using a multiple item domain-specific questionnaire or an objective measurement tool that can distinguish between postures [37, 40].

#### Strengths and limitations

This study had several strengths including the concordant measurement period for each of the tools (same 7 days), the rigour that was applied during the sampling process and a sample of individuals stratified by their physical activity level, which improves the generalisability of the findings.

Limitations included the small sample size, especially for analyses of change that resulted from invalid accelerometer data and incomplete self-report questionnaires. The required sample size for both study components, to give a 95% confidence interval of approximately +/−0.34 standard deviations [27], is 100 individuals, which we did not achieve due to loss of data following cleaning. This limitation was also found by Prince et al. who recorded inconsistencies and time lags between the number of days that were assessed by the self-report and objective physical activity measures [17]. However the current study was larger than previous analyses of the validity of GPAQ [22].

The gold standard for the measurement of free living energy expenditure is doubly labelled water (DLW) [43], but this is an expensive tool to implement, requires trained professionals to manage its implementation and has high levels of both participant and researcher burden [44]. Therefore in this study, an accelerometer has been used as a viable, practical and acceptable alternative. Accelerometers have previously been shown to be reliable and validated against DLW, making the accelerometer an acceptable criterion device for use in this study [43, 44].

Our review of previous research indicated that the Actigraph GT3X was the most appropriate commercially available accelerometer for our study. This particular model has been shown to be a valid and reliable tool, approved for use in clinical and epidemiological studies to measure PA in free living populations [45] and found to have a good level of inter-instrument reliability [46]. In addition, Actigraph has previously been reported as the “only commercially available accelerometer that has repeatedly been shown to significantly correlate with DLW-derived energy expenditure” [44].

However, accelerometers have their limitations. For example, an accelerometer does not measure water based activities or non-ambulatory activities such as cycling, so they may therefore underestimate MPVA [47]. On the other hand, self-reported measurement methods have their own limitations as they are prone to biases such as recall and social desirability [48]. Therefore, it may be appropriate to propose the use of both a subjective and an objective measurement tool, whenever possible, to obtain an assessment of PA type, intensity, duration, context and location [42]. Implementing a composite PA measurement strategy would ensure that the inadequacies of self-report and objective tools individually are compensated for by the other.

## Conclusion

In conclusion, the present study adds important new data on the validity of the widely-used GPAQ in a high-income country for estimating PA and SB levels as well as evaluating change. Overall the results suggest that the self-report GPAQ may be used appropriately to estimate levels of MVPA and monitor change in MVPA in a population sample and thus to assess the effectiveness of PA interventions on a community or population level. However present results suggest the GPAQ is not a valid measure of time spent in SB or change in SB over time in healthy free living adults.

## Abbreviations

CLC: Claire L Cleland
CCG: Connswater community greenway
CI: Confidence intervals
GPAQ: Global physical activity questionnaire
HSC: Health and social care
JFS: James F Sallis
MEC: Margaret E Cupples
MAT: Mark A Tully
MET: Metabolic equivalent
Mins: Minutes
MVPA: Moderate-to-vigorous physical activity
NCDs: Non-communicable diseases
NICE: National Institute for Health and Care Excellence
PA: Physical activity
PARC: Physical activity and the rejuvenation of connswater
R&D: Research & development
RFH: Ruth F Hunter
SB: Sedentary behaviour
SD: Standard deviation
SED: Socio-economically disadvantaged
T1: Time point 1
T2: Time point 2
UK: United Kingdom
UKCRC: UK Clinical Research Collaboration
USA: United States of America
WHO: World Health Organisation.
